# PAPSS2‐Related Brachyolmia: Clinical and Radiographic Features and Growth Hormone Therapy of One Chinese Case

**DOI:** 10.1002/ccr3.70788

**Published:** 2025-09-07

**Authors:** Wenjun Long, Xiaoping Luo

**Affiliations:** ^1^ Department of Pediatrics Tongji Hospital, Tongji Medical College, Huazhong University of Science and Technology Wuhan Hubei China

**Keywords:** asymmetric short stature, brachyolmia, growth hormone, PAPSS2, skeletal dysplasia

## Abstract

Brachyolmia type 4 (BCYM4, OMIM 612847) is a rare skeletal dysplasia characterized by mild epiphyseal and metaphyseal abnormalities. We report a Chinese boy with brachyolmia caused by a novel compound heterozygous mutation in the *PAPSS2* gene. Prenatal ultrasound revealed shortened long bones, and his birth length was markedly reduced (45 cm, −3.11 SD). Clinical and radiographic findings were consistent with brachyolmia, and genetic analysis confirmed the diagnosis of BCYM4 at 2 years and 9 months old. During follow‐up, the patient exhibited progressive growth retardation. Under pediatric orthopedics supervision, growth hormone (GH) therapy was initiated to ameliorate his short stature since 5 years and 6 months old. Over a 2‐year and 3‐month treatment period, GH therapy significantly improved his growth velocity, with his height increasing from −5.02 SD to −3.87 SD. Notably, severe growth restriction was evident as early as 25 weeks' gestation, and spinal radiographs demonstrated persistent skeletal abnormalities. This case expands the phenotypic spectrum of BCYM4 and provides evidence supporting the efficacy of GH therapy in improving growth outcomes in patients with skeletal dysplasia‐associated short stature.

**Summary**

- BCYM4 is a skeletal dysplasia caused by a PAPSS2 gene mutation.
- A Chinese boy with BCYM4 had prenatal short long bones and progressive growth retardation after birth.
- GH therapy improved his height (−5.02 SD to −3.87 SD) over 2 years and 3 months.

## 1 Introduction

Brachyolmia 4 with mild epiphyseal and metaphyseal changes (BCYM4), also known as spondyloepimetaphyseal dysplasia Pakistani type, is an autosomal recessive inherited skeletal dysplasia caused by PAPSS2 mutation. It is characterized by short‐trunk stature and normal facial features and intelligence. The most common clinical features include short stature, spinal curvature, rectangular vertebrae, irregular endplates, intervertebral disc narrowing, and mild abnormalities in the bone epiphyses and metaphyses. It was first reported by Ahmad et al. in 1998 [1]; there have been only a few dozen reported cases worldwide. The clinical and radiographic characteristics of these cases have been extensively described, showing significant heterogeneity in clinical and imaging manifestations as well as gene mutations [2, 3, 4]. The PAPSS2 gene encodes the human PAPS (3′‐phosphoadenosine 5′‐phosphosulfate) synthase 2, which produces PAPS through sulfotransferases. PAPS is a universal sulfate donor required for the sulfation of various biomolecules, including glycosaminoglycans, proteins, steroid hormones (e.g., DHEA [dehydroepiandrosterone]), and various exogenous compounds (e.g., drugs). Mutations in the PAPSS2 gene may affect the skeletal and endocrine systems [5]. BCYM4 reported in the literature can be categorized into three major types: those primarily affecting the spine with relatively mild effects on the bone epiphyses and metaphyses [2, 4, 6, 7]; those affecting both the spine and bone epiphyses and metaphyses, Pakistani‐type spinal dysplasia [1, 3]; and those with combined skeletal and endocrine manifestations as androgen excess and concurrently very low serum DHEAS [8, 9].

The clinical heterogeneity of BCYM4 is apparent, and due to the limited data of reported cases, it is difficult to draw accurate conclusions regarding the incidence rate, genotype, and phenotype correlation. In this report, we describe the clinical and radiographic manifestations of a Chinese boy with BCYM4 resulting from compound heterozygous mutations in the PAPSS2 gene inherited from both parents, in order to enrich the case resources. The child showed growth retardation during fetal development, with significantly low birth length. Typical skeletal imaging findings suggested skeletal dysplasia, and genetic analysis confirmed the diagnosis. After growth hormone therapy, the child's height increased rapidly and the degree of scoliosis remained stable with race. This report confirms the effectiveness of growth hormone treatment for skeletal abnormality‐induced short stature. The clinical data and gene analysis results of the boy are summarized as follows.

## 2 Case History/Examination

**FIGURE 1:**
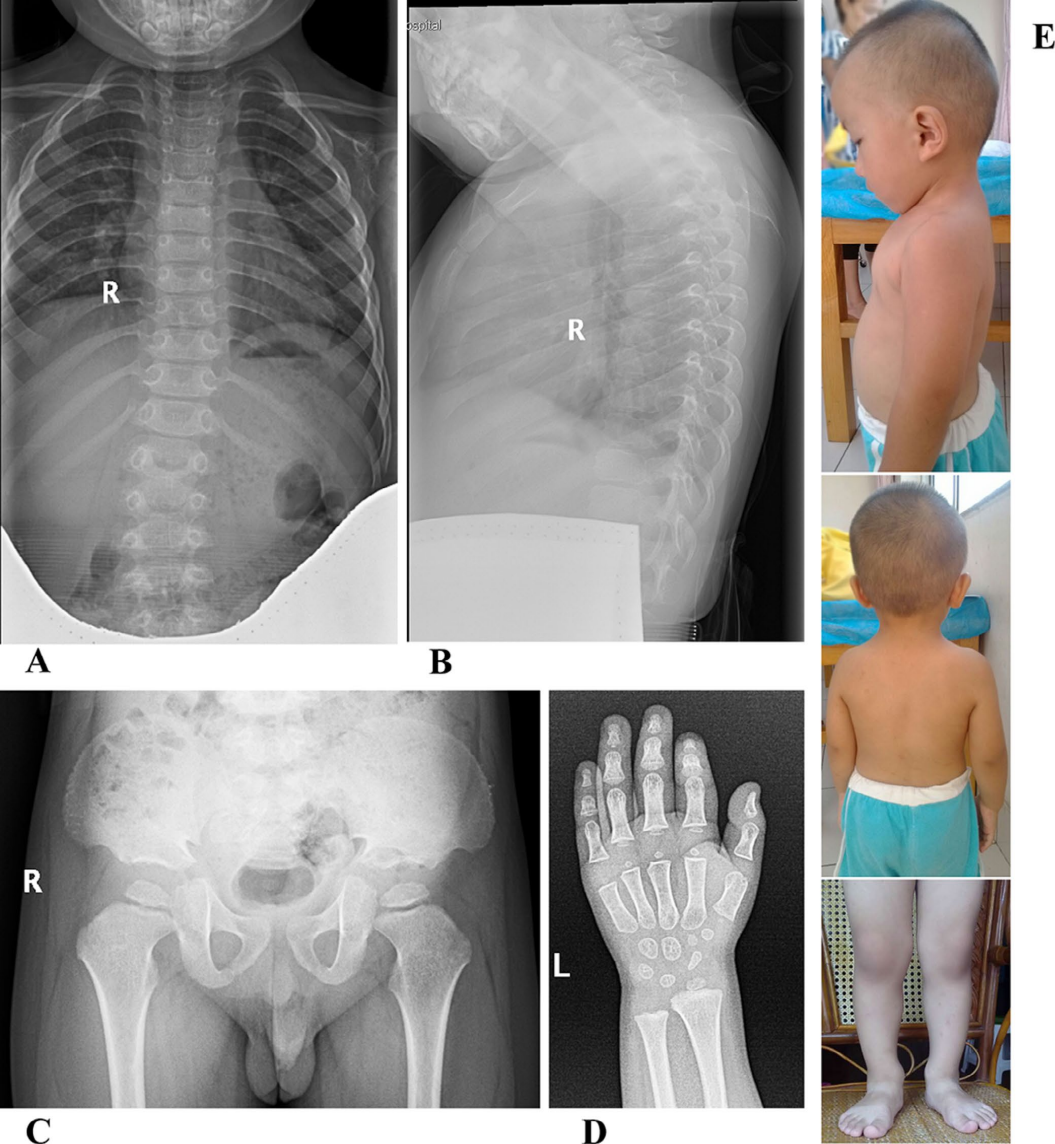
Clinical and radiographic manifestations of the child at the age of 2 years and 9 months. (A, B) Anterior and lateral views of the spine. (C) Anterior view of the hip. (D) Anterior view of the hand. (E) Clinical features of the child.

The proband, a 2‐year and 9‐month‐old boy, visited our outpatient clinic with the complaint of short stature. He had normal language and intellectual development. Short neck, slightly enlarged ankle joint, flat feet, and enamel dysplasia were noticed. His length was 81.4 cm (−3.92 SD) [10] and sitting height 52 cm. The body proportion (sitting height/(height minus sitting height)) was 1.77 (the ratio of sitting height to leg length of normal Chinese boys at 2.5 years old is 1.47, and at 3 years old is 1.41), indicating that the boy was disproportional for short stature. The head was relatively large (head circumference was 50 cm, 0.50 SD). The levels of alkaline phosphatase, calcium, phosphorus, and thyroid hormone were normal. The urine toluidine blue test was negative. Radiographs showed the bone age was 2.2 years old (TW III), and irregular endplates, narrow intervertebral spaces, rectangular pyramids, and slight scoliosis of the spine (7.4°) (Figure 1).

Short long bones were noticed at 25 weeks of gestation through ultrasound, showing that the femoral diameter was 35 mm (the average femoral diameter of normal Chinese fetus at 25 weeks of gestation is 43 mm), and the humerus length was 33 mm (the average humerus length of normal Chinese fetus at 25 weeks of gestation is 41 mm). The boy was born at 36 weeks + 3 days, and his birth weight was 2.75 kg (−1.51 SD), birth length 45 cm (−3.11 SD). At 1 year of age, he was 68.5 cm (−3.01 SD) and 76 cm (−3.69 SD) at 2 years old. The boy is born to a non‐consanguineous couple. None of the patient's grandparents or parents' siblings have short stature (adult male < 160 cm, female < 150 cm). The patient's full sister has an adult height of 155 cm (−1.04 SD). The parents' heights are 171 cm (−0.28 SD) and 150 cm (−1.96 SD), respectively, with the patient's target height being 166.5 ± 4 cm (−1.03 SD). The parents show no apparent physical abnormalities, and the patient demonstrates normal intellectual and motor development with no remarkable medical history.

## 3 Genetic Findings

**FIGURE 2:**
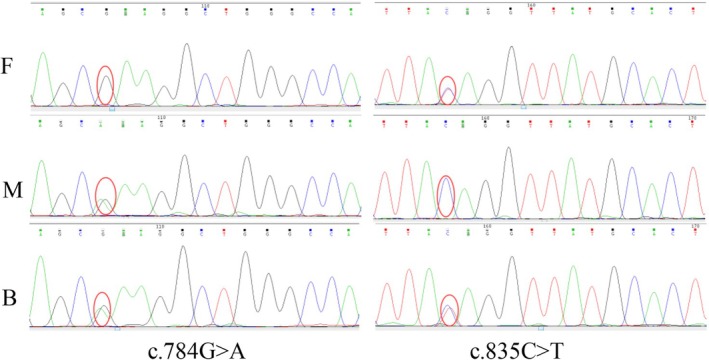
Sequence analysis of the child and his parents.

The diagnosis of spondyloepimetaphyseal dysplasia was made on clinical and radiological features. With the informed consent of parents and children, genetic testing by the first generation sequencing and Sanger sequencing identified a novel missense variant c 784G>A (p.E262K) from his mother and a novel missense variant c 835C>T (p.Q279X) from his father (Figure 2). According to the guidelines of the American College of Medical Genetics and Genomics (ACMG) in 2015, the variant from the mother is likely pathogenic and the variant from the father is pathogenic [11].

## 4 Methods

### 4.1 Genetic Analysis

With the consent of the boy's parents, genomic DNA was extracted from a venous blood sample. The DNA library was prepared by the DNA sample prep reagent set (MyGenostics, Beijing). 221 exons known to be associated with skeletal disorders were captured using the GenCap WES capture kit (MyGenostics, Beijing) and deep sequenced on the Illumina HiSeq X ten platform (Illumina, California). Variants were identified by GATK and annotated with ANNOVAR, and were further associated with multiple databases, such as 1000 Genomes, ESP6500, dbSNP, EXAC, Inhouse (MyGenostics), HGMD, and predicted by SIFT, PolyPhen‐2, MutationTaster, GERP++. Sanger sequencing was performed to confirm the potentially pathogenic variants. The identified variant was classified according to the American College of Medical Genetics (ACMG) criteria.

## 5 Conclusion and Results

**FIGURE 3:**
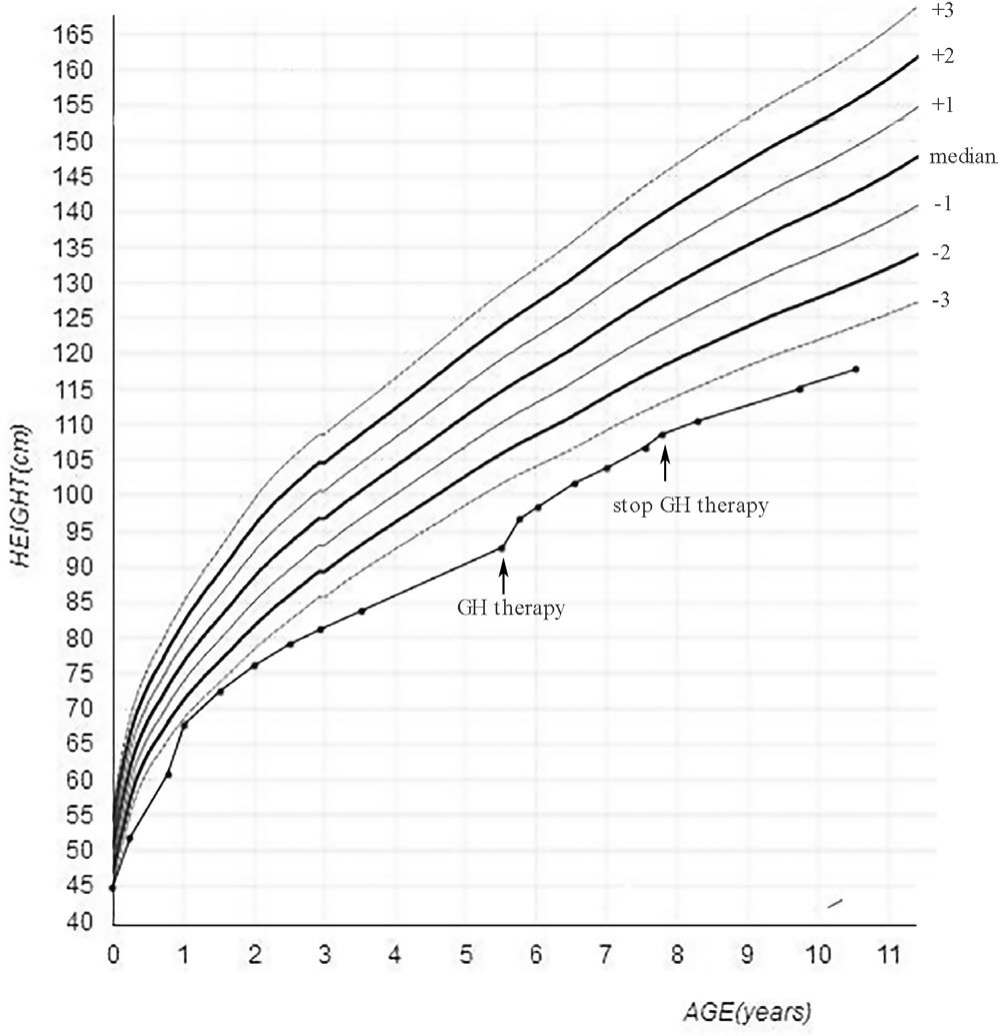
Growth curve of the child from birth to 10 years and 6 months old.

**FIGURE 4:**
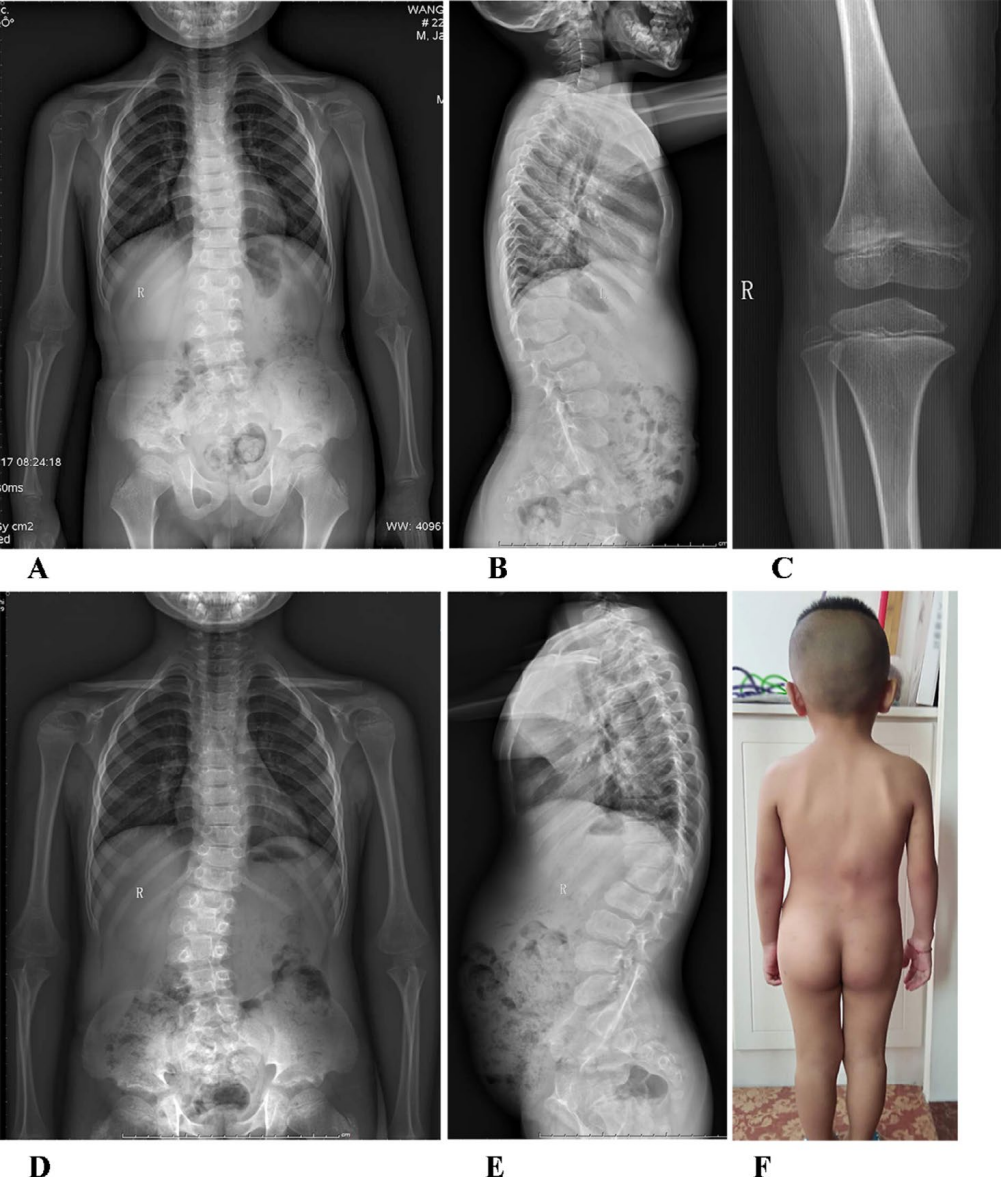
Clinical and radiographic manifestations of the child before growth hormone treatment at the age of 5 years and 6 months and stopping growth hormone treatment at the age of 7 years 9 months. (A, B) Anterior and lateral views of the spine at the age of 5 years and 6 months. (C) Anterior view of the knee at the age of 5 years and 6 months. (D, E) Anterior and lateral views of the spine at the age of 7 years and 9 months. (F) Clinical graph of the child at the age of 7 years and 9 months.

At the age of 5 years and 6 months the height was 93.3 cm (−5.02 SD) (Figure 3), and the ratio of sitting height to leg length was 1.55 (the ratio at age of 5.5 years was 1.27). He showed back pain and knee joint pain after long walking. Radiographs found that the bone age was 5.0 years and the degree of scoliosis was 13.7° (Figure 4A–C). The level of IGF‐1 concentration was 103 ng/mL (average level of normal Chinese children with same age 97 ± 37 ng/mL), and the level of IGFBP3 concentration was 3700 ng/mL (average level of normal Chinese children with same age 2628 ± 1789 ng/mL). The spine showed wedging of the first lumbar vertebral body.

According to literature reports, the final height of patients with BCYM4 ranges from −2.1 SD to −7.0 SD (including only studies with SD data) [1, 2, 3, 4, 6, 7, 8, 12]. The patient's height was −3.92 SD at the initial visit at 2 years and 9 months. The parents initially hoped that their child might be a mildly affected case, while also having concerns about the cost, efficacy, and safety of growth hormone therapy. After nearly 3 years of continuous monitoring showing progressive growth retardation, the parents decided to initiate growth hormone treatment (0.1 IU/kg.d)when the child was 5 years and 6 months old (−5.02 SD). After being treated with growth hormone, the linear growth of the child accelerated, from about 4.5 cm/y to 8.2 cm/y (−4.12 SD at 6 years and 6 months old) in the first year and 4.9 cm/y (−4.09SD at 7 years and 6 months old) in the second year (Figure 3). During the treatment with growth hormone, the level of IGF‐1 was within the normal range. After 6 months of growth hormone treatment (6 years old), the degree of scoliosis of the child progressed to 22.2°. With the assistance of orthopedic and rehabilitation doctors, the treatment with a brace was performed simultaneously with growth hormone. The degree of scoliosis was 22.4° and 22.9° after 9 months (6 years and 9 months old) and 18 months (7 years and 6 months old) of brace (Figure 4).

At the age of 7 years and 9 months old, the height was 108.6 cm (−3.99SD), and the ratio of sitting height to leg length was 1.19 (the ratio of normal Chinese boys aged 7.5 years old was 1.19) (Figure 4D–F). The level of IFG‐1 concentration was 188 ng/mL (average level of normal Chinese children with same age 170 ± 58 ng/mL), and the level of IGFBP3 concentration was 5150 ng/mL (average level of normal Chinese children with same age 3329 ± 815 ng/mL). The family stopped growth hormone for economic reasons. The parents refused to perform DHEAS and related endocrine tests, and the child did not show any signs of sexual development during follow‐up. At the age of 10 years and 6 months, the patient was followed up by telephone. The height was 118 cm (−3.94 SD), and the degree of scoliosis was 40° with a brace. The patient was ready for surgery to treat the scoliosis.

## 6 Discussion

Papss2 mainly appears in growth plate cartilage, and mutations within the PAPSS2 genes are responsible for skeletal disorder [13]. Research has shown that mutations in the PAPSS2 gene can cause brachyolmia type 4 with mild epiphyseal and metaphyseal changes. There are currently fifty more reported cases worldwide, distributed in Turkey, Syria, Lebanon, Kurdistan, Japan, Korea, Northern Europe, Africa, and China [1, 2, 3, 4, 6, 7, 8, 12].

Brachyolmia is a heterogeneous genetic skeletal dysplasia characterized by short‐trunk stature, rectangular vertebral bodies with irregular endplates, and narrow intervertebral discs, and usually accompanied by mild long bone abnormalities. We reported a Chinese boy of BCYM4 caused by two mutations in the PAPSS2 gene. Short limb length was found in the second trimester by prenatal ultrasound, which is consistent with the 8 of 18 patients identified to have short long bones on prenatal scans around 20 weeks' [4]. It suggests that fetal skeletal abnormalities discovered during pregnancy should be considered for brachyolmia. However, the prenatal ultrasound did not show bowed legs, which is inconsistent with the report by Handa et al. [7]. Our patient had significant short stature during the neonatal period, and the change in body proportion was consistent with the pattern of growth over time; while the limbs are relatively short initially, spine shortening becomes more obvious later, as reported by Bownass et al. [4]. Consistent with other reports [1, 2, 3, 4, 8], our case presented with scoliosis, which became more pronounced over time. The other phenotypes of the patient are similar to previous reports, with normal facial appearance and intelligence, enlarged ankle joints, flat feet, mild back and knee pain, without abnormal gait, genu varus, or valgus.

The final height of patients reported in different literatures ranges from −2.1 SD to −7 SD (only include those with SD data). In our case, the patient's short stature progressively worsened, reaching −5.02 SD at 5 years and 6 months old, accompanied by the appearance of scoliosis. Although a significant body of literature suggests that growth hormone does not increase the risk of progression of the scoliosis, the need for bracing, or surgery among those with idiopathic short stature [14, 15, 16], we decided to administer growth hormone treatment under close monitoring. The patient's linear growth rate increased, and over a period of 2 years and 3 months, his height increased to −3.99 SD, indicating the effectiveness of growth hormone therapy for height improvement. However, it should be noted that the height growth rate was higher in the first year but significantly slowed down in the second year. Clinical observations indicate that the most significant effects of growth hormone therapy typically occur during the first year of treatment. Long‐term efficacy may be influenced by various factors, with the body's natural growth process being a crucial element. We hypothesize that as the patient's height growth progressively approaches the expected rate of their natural growth process, the growth rate will correspondingly slow down. This phenomenon may be related to changes in growth plate sensitivity to growth hormone. Previous literature reported that growth hormone treatment was ineffective for this condition [7]. In this case, growth hormone therapy demonstrated certain efficacy, which may be related to the patient's growth velocity characteristics. The patient's height was −3.11 SD at birth, and treatment was initiated when the height decreased to −5.02 SD, resulting in improvement of progressive short stature. Notably, literature reports [7] indicate that the patient's height remained relatively stable around −2.0 SD before and after growth hormone treatment. Based on these observations, we hypothesize that growth hormone may be effective for patients with declining growth velocity, while potentially having a limited effect on patients with stable growth rates.

After 6 months of growth hormone treatment, the child's scoliosis progressed by 8.5°. With brace‐assisted treatment, we continued growth hormone (GH) therapy for the child, during which the degree of scoliosis remained stable. However, after discontinuation of GH therapy, despite the patient's adherence to brace treatment and a decrease in growth rate observed at 2 years and 9 months (10 years and 6 months old) post‐cessation, the scoliosis still progressed to 40°, ultimately requiring surgical intervention. This clinical course suggests that, in this particular case, the progression of scoliosis may not be directly associated with GH administration or growth rate. Additionally, growth hormone treatment did not accelerate this boy's bone age progression.

The imaging findings of this case were generally consistent with previous reports, including rectangular vertebral bodies, irregular endplates, narrow intervertebral spaces, absence of rib calcification, short femoral neck, and no early‐onset osteoarthritis in the proximal interphalangeal joints. There were variations in bone age among different literature sources, but most of them were within the normal range. The bone age of this case is within the normal range. Additionally, the patient also exhibited a wedge‐shaped compression of the L1 vertebra, which has not been previously reported in the literature. This case provides a valuable addition to the phenotypic spectrum of BCYM4. The PAPSS2 gene mutation in this patient is a compound heterozygous mutation inherited from the parents and appears to be a novel mutation. There is currently no clear correlation between different literature sources regarding phenotypes and genotypes, which may be due to the limited number of cases available.

The PAPSS2 encodes the enzyme PAPS synthase 2, which generates PAPS through sulfur transferase. PAPS serves as the sulfate donor required for the sulfation of DHEA to DHEAS by DHEA sulfotransferase (SULT2A1). Impaired sulfation of DHEA leads to an increased pool of DHEA available for conversion to active androgens, resulting in androgen excess; clinically manifesting with androgen excess include accelerated bone maturation, premature pubarche, and polycystic ovary syndrome. In this case, the child did not undergo DHEA testing during the follow‐up period, but there was no evidence of advanced bone age, premature pubarche, or other signs of androgen excess.

The case involves a child with intrauterine anomalies and significantly lower birth length than normal values. The child had not received medical treatment before, and at the age of 2 years and 9 months, he was diagnosed with disproportionate short stature due to small stature and a large head with a short neck. Radiographic examination revealed characteristic spinal images suggesting this condition, and ultimately genetic testing confirmed the diagnosis. Therefore, it is recommended to perform radiographic examinations for children with disproportionate short stature, as genetic testing can provide a definitive diagnosis. Growth hormone therapy has a certain effect on promoting height growth in affected children, but its efficacy is limited compared to children with growth hormone deficiency. The child's spinal abnormalities worsened with age and eventually required surgical treatment.

## Author Contributions

**Wenjun Long:** data curation, investigation, project administration, writing – original draft. **Xiaoping Luo:** conceptualization, methodology, writing – review and editing.

## Ethics Statement

This study was approved by the Ethics Committee of Tongji Hospital, Tongji Medical College, Huazhong University of Science and Technology (Approval Number: TJ‐IRB202402040). All study procedures were conducted in accordance with the tenets of the Declaration of Helsinki.

## Consent

The patient and his parents provided written informed consent to publish the date.

## Conflicts of Interest

The authors declare no conflicts of interest.

## Data Availability

Data available within the article.
